# Lessons learned from additional research analyses of unsolved clinical exome cases

**DOI:** 10.1186/s13073-017-0412-6

**Published:** 2017-03-21

**Authors:** Mohammad K. Eldomery, Zeynep Coban-Akdemir, Tamar Harel, Jill A. Rosenfeld, Tomasz Gambin, Asbjørg Stray-Pedersen, Sébastien Küry, Sandra Mercier, Davor Lessel, Jonas Denecke, Wojciech Wiszniewski, Samantha Penney, Pengfei Liu, Weimin Bi, Seema R. Lalani, Christian P. Schaaf, Michael F. Wangler, Carlos A. Bacino, Richard Alan Lewis, Lorraine Potocki, Brett H. Graham, John W. Belmont, Fernando Scaglia, Jordan S. Orange, Shalini N. Jhangiani, Theodore Chiang, Harsha Doddapaneni, Jianhong Hu, Donna M. Muzny, Fan Xia, Arthur L. Beaudet, Eric Boerwinkle, Christine M. Eng, Sharon E. Plon, V. Reid Sutton, Richard A. Gibbs, Jennifer E. Posey, Yaping Yang, James R. Lupski

**Affiliations:** 1 Department of Molecular and Human Genetics, Baylor College of Medicine, Houston, TX 77030 USA; 2 Institute of Computer Science, Warsaw University of Technology, 00-665 Warsaw, Poland; 3 Norwegian National Unit for Newborn Screening, Women and Children’s Division, Oslo University Hospital, 0424 Oslo, Norway; 4 CHU Nantes, Service de Génétique Médicale, 9 quai Moncousu, 44093 Nantes, CEDEX 1 France; 5 Atlantic Gene Therapies, UMR1089, Nantes, France; 6 Institute of Human Genetics, University Medical Center Hamburg-Eppendorf, 20246 Hamburg, Germany; 7 Department of Pediatrics, University Medical Center Hamburg-Eppendorf, 20246 Hamburg, Germany; 8 Texas Children’s Hospital, Houston, TX 77030 USA; 9 Baylor Genetics, Baylor College of Medicine, Houston, TX 77030 USA; 10 Jan and Dan Duncan Neurological Research Institute, Texas Children’s Hospital, Houston, TX 77030 USA; 11 Department of Pediatrics, Baylor College of Medicine, Houston, TX 77030 USA; 12 Texas Children’s Hospital Center for Human Immuno-Biology, Houston, TX USA; 13 Human Genome Sequencing Center, Baylor College of Medicine, Houston, TX 77030 USA; 14 Human Genetics Center, University of Texas Health Science Center at Houston, Houston, TX 77030 USA; 15 Texas Children’s Cancer Center, Texas Children’s Hospital, Houston, TX 7703 USA; 16 Baylor-Hopkins Center for Mendelian Genomics, Baltimore, MD USA; 17 Department of Molecular and Human Genetics, Baylor College of Medicine, One Baylor Plaza, Room 604B, Houston, TX 77030-3498 USA; 18 Present Address: Department of Pathology and Laboratory Medicine, Indiana University School of Medicine, 350 W. 11th Street, Indianapolis, IN 46202 USA

## Abstract

**Background:**

Given the rarity of most single-gene Mendelian disorders, concerted efforts of data exchange between clinical and scientific communities are critical to optimize molecular diagnosis and novel disease gene discovery.

**Methods:**

We designed and implemented protocols for the study of cases for which a plausible molecular diagnosis was not achieved in a clinical genomics diagnostic laboratory (i.e. unsolved clinical exomes). Such cases were recruited to a research laboratory for further analyses, in order to potentially: (1) accelerate novel disease gene discovery; (2) increase the molecular diagnostic yield of whole exome sequencing (WES); and (3) gain insight into the genetic mechanisms of disease. Pilot project data included 74 families, consisting mostly of parent–offspring trios. Analyses performed on a research basis employed both WES from additional family members and complementary bioinformatics approaches and protocols.

**Results:**

Analysis of all possible modes of Mendelian inheritance, focusing on both single nucleotide variants (SNV) and copy number variant (CNV) alleles, yielded a likely contributory variant in 36% (27/74) of cases. If one includes candidate genes with variants identified within a single family, a potential contributory variant was identified in a total of ~51% (38/74) of cases enrolled in this pilot study. The molecular diagnosis was achieved in 30/63 trios (47.6%). Besides this, the analysis workflow yielded evidence for pathogenic variants in disease-associated genes in 4/6 singleton cases (66.6%), 1/1 multiplex family involving three affected siblings, and 3/4 (75%) quartet families. Both the analytical pipeline and the collaborative efforts between the diagnostic and research laboratories provided insights that allowed recent disease gene discoveries (*PURA*, *TANGO2*, *EMC1*, *GNB5*, *ATAD3A*, and *MIPEP*) and increased the number of novel genes, defined in this study as genes identified in more than one family *(DHX30* and *EBF3).*

**Conclusion:**

An efficient genomics pipeline in which clinical sequencing in a diagnostic laboratory is followed by the detailed reanalysis of unsolved cases in a research environment, supplemented with WES data from additional family members, and subject to adjuvant bioinformatics analyses including relaxed variant filtering parameters in informatics pipelines, can enhance the molecular diagnostic yield and provide mechanistic insights into Mendelian disorders. Implementing these approaches requires collaborative clinical molecular diagnostic and research efforts.

**Electronic supplementary material:**

The online version of this article (doi:10.1186/s13073-017-0412-6) contains supplementary material, which is available to authorized users.

## Background

Applications to clinical practice of whole exome sequencing (WES) and whole genome sequencing (WGS) technologies and the computational interpretation of rare variants in genome data have been revolutionary, allowing conclusions to diagnostic odysseys and enabling molecular diagnoses for thousands of patients [1–7]. Moreover, such genome-wide assays have enabled insights into multi-locus contributions to disease [8]. Recent reports document an initial ~25–30% rate of molecular diagnosis in known disease genes for patients referred for exome sequencing and interpretation [3, 5, 9–12]. The remaining undiagnosed individuals may represent: (1) limitations in concluding a molecular diagnosis using the current experimental and analytical methods of clinical genomics practice; or (2) our limited understanding of the genetics of human disease. Collaboration between the clinical, clinical molecular diagnostic, and research communities may optimize discovery of disease genes, considering the rarity of specific genetic disorders [13–17].

We performed a pilot study for systematic transfer of molecularly “unsolved” exomes from the clinical environment to a research setting, in order to potentially fuel human genetic disease gene discovery. The WES data from 74 probands for whom clinical singleton WES did not reveal a secure molecular diagnosis were augmented with WES from additional family members, where available. Additional bioinformatics filters, database resources, and interpretive analyses were implemented, leveraging systematic studies emerging from the research laboratory. A likely disease contributory gene and potential molecular diagnosis (i.e. known disease gene or novel gene identified in more than one family) was identified in 36% of the probands, and a candidate gene finding (i.e. identified in a single family) was identified in 15% of patients. This experience and resulting findings offer the opportunity to systematically compare different but complementary approaches to optimize molecular diagnostic yield. Several novel gene discoveries (*PURA*, *TANGO2*, *EMC1*, *GNB5*, *ATAD3A*, *MIPEP*) [18–23] were facilitated by this collaborative and systematic clinical/research laboratory approach, and additional novel disease genes were found in multiple families (*DHX30*, *EBF3*), together highlighting different genetic contributions to pathogenicity [24–26].

## Methods

### Recruitment of non-diagnostic clinical exome cases into research

Probands, whose DNA had been analyzed at Baylor Genetics (BG) laboratory for clinical diagnostic WES, and for whom a molecular diagnosis (defined as a pathogenic or likely pathogenic variant according to American College of Medical Genetics and Genomics [ACMG] guidelines) was not achieved at the time of initial reporting, were classified as “unsolved” and enrolled into the study [3, 27]. For this pilot study, a total of 74 unsolved clinical WES cases, analyzed by the clinical genomics laboratory between April 2012 and April 2014, were enrolled in research between July 2013 and March 2015. Enrollment proceeded in serial order with no specific inclusion or exclusion criteria, other than the inability to achieve a molecular diagnosis in the clinical genomics laboratory and parents consenting to research analyses. Depending on the clinical situation and individual availability, additional family members (e.g. parents or affected siblings) were also enrolled.

Our pilot study consisted of 74 cases including 63 trios, four quartets, one multiplex family involving three affected siblings, and six singleton cases for which parental samples were unavailable. Prior diagnostic work-up was variable from case to case based on the referring physician’s differential diagnosis (e.g. single gene testing, enzyme assays, array comparative-genome hybridization) and included a proband-only WES in all cases. Mitochondrial DNA sequencing was performed for all cases undergoing clinical WES through December 2014. Detailed clinical phenotype data were collected and entered into PhenoDB [14, 28], after contacting the families/patients to obtain informed consent, and did not influence the choice of cases. However, we retrospectively analyzed the phenotypic features of this cohort and found that developmental delay/intellectual disability (DD/ID) was the most prevalent phenotype in our pilot study, consistent with the nature of cases referred for clinical WES [3, 5]. Our phenotypic analysis revealed 59 cases with syndromic DD/ID and one with non-syndromic DD/ID. Additionally, 14 other phenotypes encountered in the 74 cases of the pilot study were metabolic, gastrointestinal, and mitochondrial abnormalities (Additional file 1: Table S1).

### Whole exome sequencing and annotation

Exome capture was performed with Nimblegen reagents using the Baylor College of Medicine (BCM) Human Genome Sequencing Center (HGSC) custom-designed capture reagent VCRome 2.1 for both clinical and research laboratory exomes. This capture reagent contains more than 196K targets and 42 Mbp of genomic regions and includes predicted coding exons from Vega, CCDS, and RefSeq. Samples are multiplexed (six-plex format) for both capture and sequencing and full-length blocking oligos were employed for hybridization to enhance on-target specificity [3, 5, 29]. Clinical WES targets the coding exons of ~20,000 genes with 130X average depth of coverage and greater than 95% of the targeted bases having >20 reads [3, 5, 29, 30]. Research WES (for additional family members) had an average depth of coverage of 95X, with >92% of the targeted bases having >20 reads. The raw sequence data were post-processed using the Mercury pipeline [31]. First, the raw sequencing data (bcl files) were converted to fastq files using Casava. Then, the Burrows-Wheeler Alignment (BWA) tool was utilized to map short reads to the human genome reference sequence (GRCh37). Finally, the recalibration and variant calling were performed using GATK [30] and the Atlas2 suite, respectively [32]. The Mercury pipeline is available in the cloud via DNANexus (http://blog.dnanexus.com/2013-10-22-run-mercury-variant-calling-pipeline/). For research cases, exome variant analyses were then independently performed in the Baylor Hopkins Center for Mendelian Genomics (BHCMG) [15] under a Research Protocol approved by the Institutional Review Board (IRB) for Human Subjects Research at BCM; this protocol enables bi-directional transfer of samples and data between the clinical and research laboratories.

### Identification of de novo single nucleotide variants (SNVs) in BHCMG

De novo variants were identified by an in-house developed software called DNM (de novo mutation)-Finder (https://github.com/BCM-Lupskilab/DNM-Finder), available upon request. Parental variants were subtracted in silico from the proband’s variants in vcf files, while incorporating read number information extracted from BAM files. Filtering was then implemented using the following criteria: (1) an alternative variant read count greater than 5 in the proband; (2) ratio of alternative variant read count to reference variant read count greater than 30% in the proband; (3) reference variant read count greater than 10 in both parents; and (4) ratio of alternative variant read count to reference variant read count less than 5% in both parents.

### SNV prioritization and filtering workflow

**Fig. 1:**
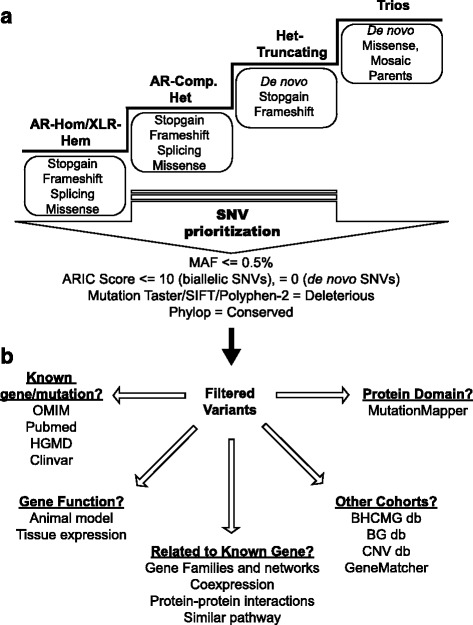
Analysis of WES data. **a** SNVs were filtered and prioritized according to specific criteria, including mode of inheritance, mutation type, variant frequency, conservation, and predictions of pathogenicity. **b** Candidate genes were further prioritized by data mining, taking into consideration gene function, expression, and networks. In addition, other cohorts were interrogated for additional families with variants in the same candidate gene. MutationMapper (http://www.cbioportal.org/mutation_mapper.jsp), *ARIC* Atherosclerosis Risk in Communities Study, *AR-Hom* autosomal recessive-homozygous, *BHCMG* Baylor-Hopkins Center for Mendelian Genomics, *BG* Baylor Genetics laboratories, *CNV* copy number variation, *Comp* compound, *db* database, *ExAC* Exome Aggregation Consortium, *Het* heterozygous, *HGMD* Human Gene Mutation Database, *MAF* minor allele frequency; *SNV* single nucleotide variant, *XLR-Hem* X-linked recessive-hemizygous

We designed a stepwise analysis workflow. This workflow consists of four major steps of analysis that examined: (1) recessive homozygous predicted loss-of-function (LOF) variants (stop-gain, frameshift indels, and splice site variants) and/or missense variants; (2) compound heterozygous LOF and/or missense variants; (3) heterozygous LOF variants, which facilitated the detection of potential truncating de novo mutations in proband-only WES cases; and (4) de novo variants and potential parental mosaic variants using trio-WES (Fig. 1a). Variants were prioritized for further study based on their minor allele frequencies (MAF; <0.5%) and the output from several prediction software algorithms that identified cross-species conservation or variant effects on protein function (Fig. 1a) [33]. Subsequently, each variant was further filtered based on additional gene and variant-level information from publicly available databases: Online Mendelian Inheritance in Man (OMIM: http://www.omim.org); PubMed; the Human Gene Mutation Database (HGMD: http://www.hgmd.cf.ac.uk); and ClinVar (https://www.ncbi.nlm.nih.gov/clinvar/). This sequential analysis eliminates the bias towards a particular mode of inheritance, mutation type, and the prior knowledge of the role of a specific gene in human disease. Additionally, this approach also integrates the fundamentals of WES analysis based on previously described SNV workflows and is compatible with ACMG guidelines for SNV filtering and classification of variant pathogenicity [3, 9, 27]. Analysis of interactomes, gene families, protein–protein interactions, co-expression data, and available literature further prioritized candidate variants (Fig. 1b). The patterns of segregation of the remaining candidate variants were analyzed by Sanger sequencing in available family members. Finally, the most promising candidate genes/variants were used to interrogate local databases and GeneMatcher [13, 14] to find additional affected cases with damaging variants in the same gene and with similar clinical phenotypes. When the BHCMG identified suspected causative variants in either newly discovered or recently published known disease genes, it was communicated to the clinical exome laboratory. Sanger confirmation of the suspected pathogenic variant was performed for all of the identified variants (confirmation rate of 100%) under the auspices of the CAP/CLIA-certified diagnostic laboratory and a formal updated clinical report was issued. Similarly, identified variants both in novel genes and in recent reports from the clinical exome laboratory were reported back to BHCMG, creating a bidirectional channel of intercommunication between research and clinical laboratories.

We also designed a parallel computational analysis of “bulk data” in order to accelerate our WES data analyses by scanning the BHCMG database for rare homozygous/heterozygous stop-gain variants culled from WES data of ~5000 research participants including our pilot study cases. In this analysis, we targeted the rare (MAF <0.5%) homozygous stop-gain variants from ~5000 research participants (including the 74 families) for each gene. Genes were then sorted according to the number of homozygous rare stop-gain variants existing in our database. Second, the genes were analyzed further according to SNV prioritization and filtering workflow as described above (Fig. 1). This approach was designed to accelerate the discovery of novel disease genes that exhibit phenotypic consequences through a loss-of-function mechanism.

### Detection of CNVs

CNV detection from WES data has been employed by different clinical laboratories to improve the molecular diagnostic rate [34, 35]. We applied several computational algorithms (CoNVex, Sanger Centre [ftp://ftp.sanger.ac.uk/pub/users/pv1/CoNVex/Docs/CoNVex.pdf], CoNIFER, and XHMM) to WES data to identify potential disease associated CNVs; these tools detect a clinically relevant intragenic CNV when at least three contiguous exons are deleted [36, 37]. Therefore, in addition to these algorithms, we developed an in-house pipeline HMZDelFinder (https://github.com/BCM-Lupskilab/HMZDelFinder) [38] to detect potential homozygous and hemizygous small intragenic deletions from WES data, including single exon “dropout alleles” that may be less robustly identified by current software.

## Results

**Fig. 2:**
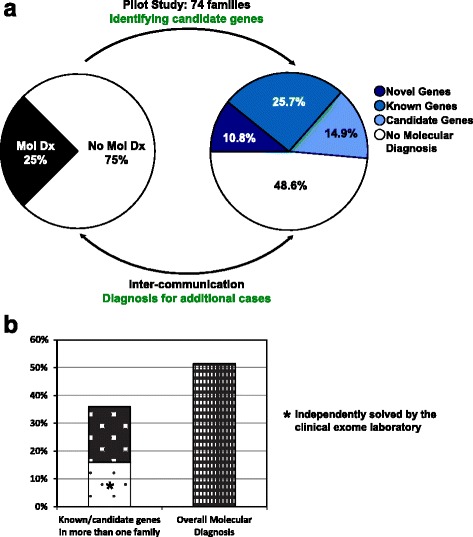
Overview of the study design and results. **a** Clinical WES cases that lacked a definitive molecular diagnosis (*left*) were eligible for recruitment into a research environment. In a pilot study of 74 families (*right*), we identified strong candidate genes for 51% (38/74) of cases. Identified variants were categorized into six major classes based on mode of inheritance and known or novel gene. **b** According to stringent criteria, a potential contributory variant was achieved in 27 of 74 (36%) cases. Of these, 12 were independently solved by the clinical exome laboratory upon reanalysis of WES data and updated literature review. When taking into account strong candidate genes identified in only a single family to date, a potential molecular diagnostic rate of 51% was achieved

The findings were sorted into three major categories: (1) known disease genes; (2) novel genes: genes with predicted pathogenic variants in two or more cases with similar phenotypes; and (3) potential candidate genes: genes with variants in a single case (Table 1). Predicted pathogenic variants in known or novel disease genes were identified in 27/74 (36%) families studied. In addition, predicted pathogenic variants in candidate genes were identified in 11/74 (15%) of cases, yielding an overall potential solved rate of 38/74 (51%) of cases. Our findings are represented by six “lessons learned” clusters: (1) de novo changes in known genes; (2) de novo changes in novel and candidate genes; (3) potential mosaicism in parents; (4) biallelic or hemizygous variants in genes known to convey recessive disease traits; (5) biallelic variants in novel and candidate recessive disease genes; and (6) blended phenotypes resulting from dual molecular diagnoses (Fig. 2a, b). Examples for each of these scenarios are presented below.

**Table 1:** Molecular diagnoses in 74 cases are represented as three major categories: known genes, novel genes and candidate genes

Inheritance	Known genes	Novel genes	Candidate genes
De novo	*CACNA1A*, *DDX3X(X2)* ^a^, *NALCN(X2)*, *NR2F1* ^a^, *ZBTB20*	*ATAD3A*, *DHX30*, *EBF3*, *EMC1*, *PURA* ^a^	*CDK20* + *HIVEP1*, *DNAH7*, *GSPT2 GUCY2C*, *MICALL2* + *SLC30A7*, *MPP4*, *SYN3*, *SYTL2*
Autosomal/X-linked Recessive	*ABCA4*, *DDX3X*, *FBXL4* ^a^, *NAA10*, *SLC13A5* ^a^ *(X2)*, *TRAPPC11*, *ZNF335* ^a^	*GNB5*, *MIPEP*, *TANGO2* ^a^	*ACOT1* ^a^, *NRXN3*, *USP19*
Other		*NA*	*NA*
UPD	*SLC1A4* ^a^		
Mosaic	*PIK3CD*		
Dual molecular diagnosis	*PMPCA + KCND3* ^a^ *POLRIC + SCNIB* ^a^		

^a^Cases independently solved by the clinical exome laboratory re-analysis

Three major categories of identified genes include 13 candidate genes identified in 11 families and 26 known or novel genes in 27 families. Note that some families had more than one molecular diagnosis (indicated by *PMPCA + KCND3*, *POLR1C + SCN1B*, *CDK20 + HIVEP1*, *MICALL2 + SLC30A7*) and some genes were identified in more than one family (indicated by “(X2)” in the table)

*NA* non-applicable

### De novo changes in known genes

Analysis of WES data from sets of parents and offspring initially yielded ~50–200 putative de novo variants per trio. Further prioritization, based upon read coverage, MAF and mutation type (non-synonymous, stop-gain, frameshift indels, and splicing variants), reduced the number of potential pathogenic de novo variants per family to ~0–5 variants per proband (Additional file 2: Figure S1). We detected de novo variants in five recently published genes (Table 1, Additional file 3: Table S2): *ZBTB20* associated with Primrose syndrome [39] (MIM 259050); *NR2F1* causing the Bosch-Boonstra-Schaaf optic atrophy syndrome [40] (MIM 615722); *DDX3X* associated with X-linked intellectual disability [41] (MIM 300958); *CACNA1A* implicated in non-fluctuating ataxia [42]; and *NALCN* associated with congenital contractures of the limbs and face, hypotonia, and developmental delay [43] (MIM 616266).

### De novo changes in novel and candidate genes

**Fig. 3:**
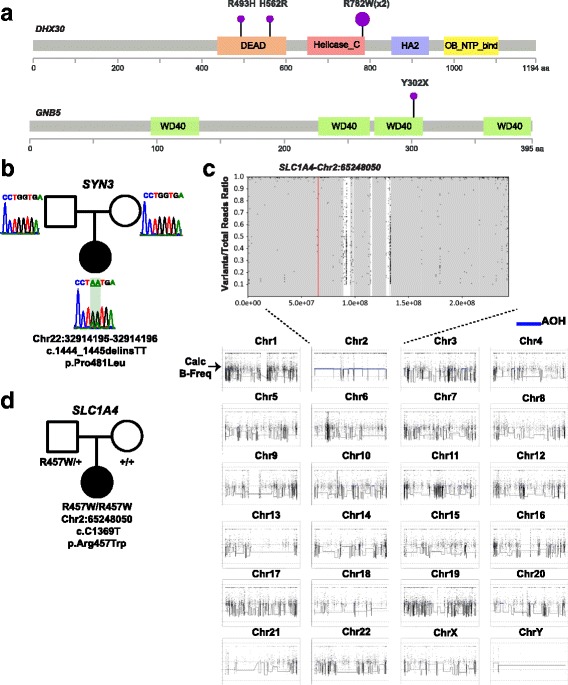
Location of *DHX30* and *GNB5* variants, dinucleotide variants culled from WES data and UPD. **a** Variants identified in *DHX30* and *GNB5* are located in specific protein domains. **b** Sanger confirmation of a de novo dinucleotide variant in *SYN3*. **c** The B-allele frequency extracted from WES data in the patient with the homozygous *SLC1A4* variant showed a single region of AOH in the genome (chromosome 2), suggestive of uniparental disomy (UPD) of chromosome 2. **d** Segregation analysis of the *SLC1A4* homozygous variant did not conform to Mendelian expectations

We further identified de novo variants in five novel and ten candidate genes (Table 1, Additional file 3: Table S2). A de novo SNV in *DHX30* c.2344C > T (p.Arg782Trp) was found in a patient with microcephaly, developmental delay/intellectual disability (DD/ID), mild cerebral volume loss, hypotonia, seizures, short stature, failure to thrive, and generalized hirsutism. Through data exchange [3, 5] with the clinical exome laboratory (Baylor Genetics) and GeneMatcher [13, 14], we found three additional unrelated participants with overlapping features, each of whom harbored a de novo SNV in *DHX30.* Interestingly, two of these participants shared the same de novo SNV c.2344C > T, occurring at a CpG dinucleotide (Fig. 3a) [44]. DEAH (Asp-Glu-Ala-His) box helicase 30 (DHX30, MIM 616423) is a member of the RNA helicase family including DHX and DDX proteins involved in DNA transcription, splicing and translation [45–47], and is highly expressed in the brain during neurogenesis [45]. Homozygous knockout of *Dhx30* in mice is lethal [45]. *DDX3X*, another member of this family, has been recognized recently to play a major role in DD/ID [41]. Additionally, *DHX37* has been proposed as a candidate gene for brain malformations [48], highlighting the importance of RNA helicases in development of the central nervous system.

*GSPT2* and *EBF3* each harbored one de novo variant in the genomes from two separate probands (Table 1, Additional file 3: Table S2). Each of these genes is located within CNV intervals identified previously in patients with DD/ID, providing additional evidence for their roles in neurodevelopmental phenotypes (Additional file 4: Supplemental text) [49–51]. Through international collaborative efforts, additional families with de novo *EBF3* variants were identified with similar phenotypes [24–26].

A de novo double nucleotide substitution in *SYN3* (c.1444_1445delinsTT; p.Pro481Leu), encoding synapsin III, was found in a proband with DD, seizures, atrophy of the cerebellar vermis, hypotonia, and a movement disorder (Fig. 3b). *SYN3* is a member of the synapsin gene family, which includes *SYN1* and *SYN2*, and plays a major role in dopamine regulation [52]. *MECP2* overexpression has been shown to result in upregulation of *SYN3* expression [53]. Taken together with the role of dopamine signaling in epileptogenesis [54], we hypothesize that the observed features of epilepsy and DD in this patient, might be associated with the de novo dinucleotide variant in *SYN3*. Finally, we identified de novo missense variants in *ATAD3A* (MIM 617183) and *EMC1* (MIM 616875) and additional collaborative efforts supported these as disease genes with both monoallelic and biallelic pathogenic variants [20, 22].

### Potential mosaicism in parents

In a multiplex family of Arab descent, in which three of five siblings have immunodeficiency, the clinical laboratory sequenced the first sibling without identifying a molecular diagnosis. Therefore, we transferred the first sibling’s WES data to BHCMG and offered WES to the other two affected family members and parents. This approach facilitated the examination of rare shared variants among three affected siblings and led to the detection of potential mosaic SNVs in the father. We identified a heterozygous variant c.1573G > A (p.Glu525Lys) in phosphatidylinositol-4,5-bisphosphate 3-kinase catalytic subunit delta (*PIK3CD*, MIM 602839) shared by the three affected siblings and inherited from their apparently healthy father. This variant was reported previously to cause an autosomal dominant form of immunodeficiency [55]. To assess possible mosaicism in the father, we calculated the ratio of variant to total reads: 6/38 (15%). This ratio significantly deviates from the expected 50% of variant to total reads (*p* value 0.003). The same calculation in the three affected siblings yielded ratios of 20/53 (37%), 37/68 (54%), and 87/204 (42%), all of which were not significantly different from the expected 50% of variant to total reads (*p* value 0.241, 0.731, and 0.164, respectively). This suggested possible mosaicism in the father, whose post-WES clinical work-up revealed mild laboratory signs of immunodeficiency (with mild reduction of NK cell cytotoxicity) compared to his severely affected children (Table 1, Additional file 3: Table S2) [56].

### Biallelic or hemizygous variants in genes known for recessive disease traits

Homozygous or compound heterozygous variants were detected in six recently described or well established disease genes: *SLC13A5* (epileptic encephalopathy, MIM 615905); *FBXL4* (mitochondrial DNA depletion syndrome 13, MIM 615471); *ZNF335* (microcephaly in a single family, MIM 615095); *SLC1A4* (spastic tetraplegia, thin corpus callosum, and progressive microcephaly, MIM 616657); *TRAPPC11* (limb girdle muscular dystrophy type 2S, MIM 615356); and *ABCA4* (cone-rod dystrophy, MIM 604116). Hemizygous variants were identified in two known X-linked disease genes: *NAA10* (Ogden syndrome, ID and long QT, MIM 300855); and *DDX3X* previously associated with X-linked ID (MIM 300958) (Table 1, Additional file 3: Table S2) [41, 57–65].

The homozygous missense variant identified in *SLC1A4*, encoding solute carrier family 1 (glutamate/neutral amino acid transporter), member 4, did not follow the expected Mendelian pattern of inheritance, since only the father carried the variant. The cSNP array data, which are performed as part of the clinical exome analysis, were integrated with calculated B allele frequency information from WES data [38] and showed absence of heterozygosity (AOH) limited to chromosome 2 and encompassing almost the entire chromosome, indicating paternal uniparental disomy with reduction of the variant to homozygosity as the responsible molecular mechanism (Fig. 3c, d).

### Biallelic variants in novel and candidate recessive disease genes

The parallel computational algorithm for detecting rare homozygous stop-gain mutations from “bulk data” (i.e. ~5000 research exomes in the BHCMG database, including this pilot study), identified a rare homozygous stop-gain mutation in *GNB5* (MIM 617182) in a study participant with DD, hypotonia, retinopathy, and Mobitz type I atrioventricular block (Fig. 3a). A homozygous splice site variant (c.249 + 3 A > G) in *GNB5* was detected in two affected siblings from the clinical diagnostic laboratory exhibiting DD/ID, nystagmus, and sinus node dysfunction. *GNB5* encodes the Gβ5 protein, a guanine nucleotide-binding protein and a member of the signal-transducing G protein β subunit family. Additional participants with pathogenic variants in this disease gene were subsequently identified [21].

Recessive variants were found in novel (*MIPEP* and *TANGO2*) [19, 23, 66] as well as candidate genes: *USP19*, *NRXN3*, and *ACOT1* (Table 1; Additional file 3: Table S2). Each of these was encountered in only one family. *USP19*, found in a proband with epileptic encephalopathy, DD, and hypotonia, encodes ubiquitin-specific protease 19, which is involved in the regulation of ataxin 3 (spinocerebellar ataxia, MIM 109150) and interacts with *DOCK7* (epileptic encephalopathy, MIM 615859) and *USP7* (autism spectrum disorder) (Additional file 2: Figure S2) [67–69]. *NRXN3* encodes a member of the neurexin family of which *NRXN1* is associated with Pitt-Hopkins-like syndrome 2 (MIM 614325) [70–72] and *ACOT1* is involved in lipid metabolism (Additional file 4: Supplemental text). Finally, we identified compound heterozygous SNVs in *MIPEP* (MIM 617228) and homozygous SNVs in *TANGO2* (MIM 616878) and found additional evidence for pathogenicity through collaboration [19, 23]. Combinations of SNVs and CNVs on alleles inherited in *trans* have been encountered in both of these genes, underscoring the potential utility of WES in identifying CNVs (including single exon deletions) as well as UPD.

### Dual molecular diagnoses in known genes

A dual molecular diagnosis [8] was established in two probands. The analysis of trio WES data revealed both compound heterozygous variants in *PMPCA* and a de novo variant in *KCND3* in an individual with DD, ataxia, epilepsy, Hirschsprung disease, and abnormal mitochondrial function (Complex I and III deficiency). In another family, quad WES analysis identified a paternally inherited heterozygous missense variant of unknown significance in *SCN1B*, the gene for Brugada syndrome 5 (MIM 612838) [73], and compound heterozygous changes in *POLR1C*, segregating in two affected siblings (Table 1, Additional file 3: Table S2, Additional file 4: Supplemental text). At the time of the initial clinical exome analysis, neither *PMPCA* nor *POLR1C* were associated with spinocerebellar ataxia and leukodystrophy, respectively. On the other hand, the clinical exome laboratory did report both *KCND3* (MIM 607346) [74] and *SCN1B* (MIM 612838) as potential contributory variants for the patients’ manifested phenotypes. Albeit, the clinical features of the patients’ phenotypes could not be completely explained by the changes in the *KCND3* and *SCN1B* genes, and both cases were subsequently enrolled into our pilot study. Our research analysis prioritized both the *PMPCA* (MIM 613036) and *POLR1C* (MIM 610060) genes along with *KCND3* and *SCN1B* as potential contributory variants prompted by recent publications, which uncovered their roles in human disease [75, 76]. Of note, *POLR1C* was described in 2011 to cause recessive Treacher Collins syndrome (MIM 248390), however the patient’s clinical features were not matching those of Treacher Collins syndrome, potentially complicated by the blended phenotype due to the dual molecular diagnosis [77].

## Discussion

We developed and implemented a workflow for: (1) the collection of new WES cases; (2) generation of additional data and analyses resources; and (3) application of further data filtering and adjuvant analysis methods to discover novel and candidate disease genes. This workflow and the data generated were used to optimize potential molecular diagnoses of Mendelian disease traits in clinical exome cases for which an initial molecular diagnosis was not achieved. This pilot study investigated 74 families and in 27 families (36%) identified a predicted damaging variant in a known or novel gene. If one considers damaging variants in candidate genes that were observed in a single family to date (11/74; 15%), the cumulative rate of potential molecular diagnoses in this pilot cohort of unsolved clinical exomes would be 51% (38/74). A particular phenotype, such as the presence or absence of ID, did not significantly influence the rate of diagnosis; i.e. a potential molecular diagnosis was achieved in 54.2% (32/59) of cases with DD/ID and 42.9% (6/14) cases without DD/ID (*p* value = 0.05; Additional file 5: Table S3).

Beyond the impact on diagnosis, potential prognostic information, and genetic counseling, several of the newly established molecular diagnoses had implications for medical management. Examples include acetazolamide treatment for *CACNA1A* mutation, surveillance for arrhythmias in *GNB5* and *TANGO2*, and mitochondrial-specific surveillance for cardiac, renal, and liver involvement in *FBXL4*. For patients with the *SCN1B* variant, current practice guidelines suggest treatment with isoproterenol as a first-line agent for electrical storm (ventricular fibrillation) and consideration of an implantable cardioverter defibrillator (ICD) to prevent sudden death [78]. In a recent study applying WES and CNV testing in clinically diagnosed primary immune deficiency diseases (PIDD), a molecular diagnosis was achieved in about 40% of unrelated probands; clinical diagnosis was revised in about half (60/110) and management was directly altered in nearly one-quarter of families based on molecular findings [56].

In about half (12/27, 44.4%) of the families, diagnosed by a known or novel disease gene, the same diagnosis was achieved independently of the research analyses as part of the routine reanalysis of WES data by the clinical exome laboratory based on recent gene discoveries (Table 1). The combined efforts of the clinical and research laboratories have led to multiple reports of novel disease genes, most of which are described in much greater phenotypic and molecular detail in independent publications (*PURA*, *TANGO2*, *EMC1*, *GNB5*, *ATAD3A*, *MIPEP*, and *EBF3*) [18–26]. Since all of these discoveries included cases from the 74 in our pilot study and can be attributed to combined efforts between the clinical and research laboratories, they are included in the overall stated molecular diagnostic yield of 36%.

Our study demonstrates that an increase in the molecular diagnostic yield can be achieved through systematic and comprehensive reanalysis of clinical exome data. Of 74 cases with non-diagnostic clinical exomes, a molecular diagnosis was achieved in 30/63 trios (47.6%), 4/6 singleton cases (66.7%), 1/1 multiplex family involving three affected siblings, and 3/4 (75%) quartet families (Additional file 6: Table S4). This increased diagnostic yield beyond clinical WES may be attributed to: (1) the rapid pace of Mendelian gene discovery; (2) the use of trio sequencing; and (3) the extensive research reanalysis and implementation of novel tools for identification of de novo and CNV variants. Vigorous collaboration between clinical and research efforts can enhance diagnostic yield and fuel novel disease gene discovery. For instance, the clinical exome laboratories’ standard operating procedure for exome analysis and variant interpretation adheres to current ACMG guidelines, which include recommendations for the reporting of pathogenic variants, likely pathogenic variants, and variants of unknown significance (VUS) in known (established) disease genes [27]. These stringent criteria do not provide for reporting of novel disease genes at the time of initial discovery, as by definition they cannot be considered pathogenic or likely pathogenic until a causal relationship between variation at a particular locus and disease has been firmly established. Thus, novel variation and novel disease genes are best studied in a research environment. In our study, the pursuit of novel disease genes for rare disease benefits from a tremendous resource: the combined exome variant dataset from over 15,000 cases referred to either the clinical exome laboratory or the BHCMG research laboratory. Research studies also support reporting of novel disease genes at the time of initial discovery, an important step toward gaining sufficient evidence to meet ACMG guidelines for reporting by a diagnostic laboratory. Additionally, as certain tools are developed and validated in a research setting and are honed to better efficiency, they are frequently translated into the clinical pipeline; GeneMatcher, developed by the BHCMG, is one such example [13, 14].

In this study, trio-WES analysis combined with de novo variant and CNV detection were invaluable for achieving an improved molecular diagnostic rate. The addition of parental samples (i.e. trio analysis) led to molecular diagnoses in 47.6% (30/63) of cases for which proband-WES was non-diagnostic. Analysis of trio-WES data supports the efficient identification of de novo and compound heterozygous variants, leading to improved analysis efficiency across all inheritance models. In an attempt to limit false-positive calls, we required that both parents have at least ten reference reads. Thus, a limitation to our analysis is that regions with poor coverage in either or both parents were parsed during variant filtering. Taking these regions/genes into account by flagging them in the bioinformatics pipeline and performing Sanger confirmation of any candidate variants at these loci may further optimize identification of candidate de novo variants. As the cost of WES continues to fall, we anticipate that trio-WES may ultimately be favored over proband-WES in the clinical setting of a sporadic suspected genetic disease.

Trio analysis also supported the use of a newly developed tool, DNM-Finder (https://github.com/BCM-Lupskilab/DNM-Finder). In our study, 27% of the pathogenic or candidate variants were de novo variants. Trio analysis can also increase the molecular diagnostic yield over that attained by studies of the proband alone. This is due, in part, to being able to more readily detect by computational filtering de novo variants (Additional file 2: Figure S1). All DNA was extracted from blood or saliva samples in this study; however, detection of mosaicism may be enhanced by including concurrent analyses of more than one lineage: ectodermal (buccal swab, hair), mesodermal, and/or endodermal (blood, saliva). The degree of mosaicism correlates with the timing of mutation, cell migration and designation during development, and tissue-specific growth profile [79]. Taking into account such considerations is important to guide the detection of mosaicism. Additionally, compound heterozygosity is more readily detected.

Analysis of all modes of inheritance and comprehensive review beyond the first identified potential pathogenic variant allowed for identification of dual molecular diagnoses in two cases (e.g. *PMPCA* and *KCND3*; *POLR1C* and *SCN1B*), consistent with previous reports of diagnoses in 5–7% of molecularly diagnosed cases [3, 5, 8, 9, 56, 80, 81]. These cases underscore the need for a systematic and comprehensive approach to exome variant analysis for all possible modes of inheritance as well as an updated literature review [12]. This is especially true of cases in which we observe apparent “phenotypic expansion” (clinical features previously unreported in association with the gene) or unexpected clinical severity [8].

Ancillary approaches to “bulk data” reanalysis were shown to enhance the molecular diagnostic rate. These include the examination of rare homozygous stop-gain SNVs, extraction of copy number information from the WES data to search for regions of absence of heterozygosity (AOH) (i.e. *ABCA4* and *FBXL4*) and assessing UPD (i.e. *SLC1A4*) and small deletion CNVs that might escape detection by CMA (Fig. 3c, Table 2, Additional file 2: Figure S3) [19, 38]. We also underscore the importance of capturing single-exon genes on WES platforms (*PURA*, *GSPT2*); these can be challenging as they are often rich in GC content, similar to the first exon of multiexonic genes [82].

**Table 2:** Lessons learned that may increase the molecular diagnostic yield from unsolved clinical exomes

Lesson Learned	Examples
A) Collaboration between research and clinical laboratories	Sharing data, open communication of findings, access to additional patients with damaging variants
B) Facilitating research collaborations including local and international efforts	GeneMatcher for the identification of unrelated affected individuals with the same novel disease
C) Ancillary approaches to enhance molecular diagnostic rate:	
1) Detection of AOH and CNVs from WES 2) Annotation of single exon genes 3) Analyze intronic variants (including those in the unfiltered vcf files) 4) Optimization of the bioinformatic filters 5) Look for *in trans* inheritance of SNVs and CNVs 6) Increase fidelity of calling dinucleotide substitutions	1) *ABCA4, SLC1A4, TANGO2, FBXL4* 2) *PURA, GSPT2* *3) TRAPPC11* 4) *ABCA4, NRXN3* 5) *TANGO2* ^a^ *, MIPEP* 6) *SYN3*
D) Ancillary approaches for non-conclusive WES:	
1) Consider dual molecular diagnoses 2) Analyze additional family members 3) Look for parental mosaicism 4) Identify homozygous stop-gain SNVs from bulk data to identify additional affected individuals	1) *PMPCA* and *KCND3; POLR1C* and *SCN1B* ^a^ 2) *SLC13A5, NAA10* 3) *PIK3CD* 4) *GNB5* ^a^
E) Consideration of different inheritance patterns and variant types at a single locus	
1) AR, AD 2) AD, CNV del 3) AR, CNV del 4) XLR, CNV dup 5) AD, Tandem repeats 6) AR, AR	*1) ATAD3A, EBF3, EMC1, GSPT2, NALCN, GUCY2C* *2) EBF3, PURA, ZBTB20* *3) NRXN3* *4) GSPT2* *5) CACNA1A* ^a^ *6) POLR1C*

^a^The molecular diagnosis in these genes had direct implications for clinical management

*AD* autosomal dominant, *AOH* absence of heterozygozity, *AR* autosomal recessive, *del* deletion, *dup* duplication, *CNV* copy number variation, *SNV* single nucleotide variants, *WES* whole exome sequencing, *WGS* whole genome sequencing, *XLR* X-linked recessive

Our study highlights the limitations of different WES data variant calling pipelines. The research laboratory opts for less stringent filtering of variants, which allows identification of intronic variants located farther from the splice site (i.e. *ABCA4*); albeit, this increases the number of variants to be investigated and consequently time taken for analysis. For instance, clinical WES did not reveal a conclusive molecular variant in a proband who presented with cone-rod dystrophy from Indian-Asian ancestry and consanguineous parents. Our research bioinformatics pipeline detected a homozygous intronic frameshift deletion, which possibly could explain the phenotype. The inherited deletion was more common in the South Asian population (seen 14 times on ExAC filtered variants). We also identified shared limitations between both pipelines, i.e. dinucleotide substitutions called as two separate variants. Our pilot study reflects the strength of collaborative efforts and iterative analyses between clinical and research teams for the optimization of computational pipelines to analyze WES data and further understand the genetic architecture underlying disease, both Mendelian disease and common/complex traits (Table 2).

This study confirms and extends our understanding of the relationship between variation at a locus and disease expression. We identified examples of genes for which different variant alleles are associated with either a dominant or a recessive disease trait (e.g. *EMC1*, *ATAD3A*, *NALCN*, and *GUCY2C*) [20, 22, 43, 83–86]. Other genes presented with recessive changes or de novo SNVs/CNVs underlying different phenotypes (e.g. *NRXN3*) for which heterozygous CNVs have previously been reported with disease [87]. Several genes were found to have de novo SNV and CNV alleles: *EBF3*, *GSPT2*, *PURA*, and *ZBTB20* [18, 23, 26, 39, 50, 51, 88–90]. This phenomenon can be exemplified by recent reports of glutamate receptor, ionotropic, delta 2 (*GRID2*, MIM 616204), which can contribute to neurodevelopmental disorders and ataxia through recessive and de novo SNVs as well as homozygous and de novo partial CNV deletions (Table 2) [91–95]. Furthermore, *CACNA1A* gene function can be altered either by de novo SNVs or trinucleotide repeat expansion (CAG) to manifest with a broad range of neurological disorders [42, 96, 97]. Yet another class of genes can present with distinct disorders due to allelic heterogeneity, e.g. *POLR1C* associated with recessive hypomyelinating leukodystrophy 11 (MIM 616494) and recessive Treacher Collins syndrome 3 (MIM 248390) [76] and illustrate allelic affinity wherein different clinical disease phenotypes are due to different alleles at the same locus [98]. In addition, compound heterozygous SNV and CNV alleles have been exemplified in other patients exhibiting *MIPEP* and *TANGO2* associated phenotypes (Table 2) [19, 23, 66]. These findings of different variant allele types and combinations thereof underscore the notion that defining potential causative alleles necessitates consideration of all variant types (SNV, indels, and CNV) and a multitude of potential genetic mechanisms (e.g. alleles causing dominant traits while other alleles cause recessive disease traits, *EMC1* and *ATAD3A* [20, 22]) and inheritance patterns while seeking answers for the genetic basis of Mendelian phenotypes.

These data dramatically illustrate how genetic disease can be driven by rare recent variants introduced into a family, further supporting the clan genomics hypothesis [99]. The clan genomics model could explain how the same gene may contribute to disease by either de novo and/or recessive SNVs and/or CNVs. De novo events arise in each generation from the failure of DNA repair or replication errors [100]. Rare de novo events with strong mutation effects (i.e. rare variants, predicted to be deleterious) may manifest as disease in the first generation [100, 101], in contrast to weaker variant alleles which require a second pathogenic allele or reduction to homozygosity in order to manifest as a trait or disease in subsequent generations (i.e. *ATAD3A*, *EMC1*, *GUCY2C*, and *NALCN* genes) [20, 22, 43, 83–86]. Remarkably, some carrier states for recessive disease may raise the susceptibility for a common, complex trait as age progresses, as demonstrated by heterozygous SNVs observed in *ABCA4*, also known as *ABCR*, (MIM 153800), *CFTR* (MIM 167800), and *LDLR* (MIM 143890) genes leading to age-related macular degeneration, pancreatitis, and familial hypercholesterolemia, respectively [63, 99, 102–107]. Taken together, the heterogeneity of phenotypes, different inheritance patterns, and different kinds of variants (SNVs or CNVs) presented in our study may lead to an enhanced understanding of a unified genetic model for human disease [99].

As the price of WES falls, trio analysis will be more efficient for sporadic traits than singleton analysis, in that it allows for detection of de novo variants and phasing of compound heterozygous variants prior to Sanger validation. This enhances detection of relevant variants in known genes and serves to catalyze novel gene discovery. Based on our study, we propose a workflow for the management of non-conclusive singleton clinical WES (Table 2, Additional file 2: Figure S4). This workflow intends to investigate the many possible scenarios that could be encountered when clinical exomes are non-productive for a specific molecular diagnosis. These include: (1) de novo missense SNVs; (2) dual molecular diagnoses; (3) multiple affected family members; (4) potential parental mosaicism; (5) in *trans* inheritance of SNVs and CNVs; (6) loosening the default parameters of bioinformatics filters for parsing variants; and (7) potential intronic unfiltered variants on variant calling files.

A study that applied WGS to 50 clinical cases that remained unsolved by genomic studies, i.e. in which probands initially had non-conclusive clinical microarrays and WES, revealed an additional molecular diagnostic yield of 42%. This 42% molecular diagnostic yield was driven mainly by de novo SNVs and CNVs impacting the coding regions [108]. In the current research reanalysis of 74 clinical exomes, by implementing WES reanalysis and augmentation with additional family members, WES achieved a similarly increased molecular diagnosis yield (36%) of unsolved exomes and even higher increased rate (51%) if potential candidate genes are considered. These pilot studies data (50 WGS versus 74 WES + reanalysis) suggest that currently WGS offers no significant advantage to WES and reanalysis when it comes to increasing molecular diagnostic yield from unsolved clinical exomes. Nevertheless, the diagnostic yield by WGS may potentially be increased further by the development of new bioinformatic algorithms to detect intronic variants or variants in regulatory regions as well as structural variations followed by further functional characterization. Future studies comparing WGS versus WES performances for diagnostic yield should use both of these techniques in parallel while also taking other parameters (coverage and cost) into account.

## Conclusion

We have demonstrated that systematic study of “unsolved clinical exomes” can provide a rich resource for Mendelian gene discovery and that reanalysis of data coupled with incorporation of additional family member WES data can improve the molecular diagnostic rate. These research studies can, in turn, provide the basis for improving interpretive algorithms for clinical WES analyses (Additional file 2: Figure S4). Our data additionally highlight the remarkable contribution of new mutation to disease including blended phenotypes resulting from dual molecular diagnoses [3, 5, 8]. Speculation based on these pilot study data suggests that if one considers the 25–30% molecular diagnostic rate achieved by initial clinical exome analyses, in combination with the 51% rate found in these pilot research studies of unsolved clinical exomes, genomic analyses by WES have the potential to identify a rare variant and gene that implicate a molecular diagnosis which could impact clinical decisions in ~ 63 (25% + [75%*51%]) to 66% (30% + [70%*51%]) or the majority of cases.

## Additional files

Additional file 1: Table S1. Summary of phenotypic features for each case. (XLSX 1227 kb)

Additional file 2: **Figures S1**, **S2**, **S3**, and **S4.** Computational *de novo* variant detection from WES data, interactome analysis of *USP19*, AOH maps for probands with *ABCA4* and *FBXL4* variants and clinical whole exome management. (PDF 3462 kb)

Additional file 3: Table S2. *De novo* and recessive variants in candidate or recently published genes identified by collaboration between BG and BHCMG. (XLSX 28 kb)

Additional file 4: Supplemental text. (DOCX 118 kb)

Additional file 5: Table S3. Diagnostic rate differs among groups of patients with different phenotypic features. (XLSX 8 kb)

Additional file 6: Table S4. Diagnostic rate in singleton cases, trios, quartets and families with multiple affected individuals. (XLSX 28 kb)

## Abbreviations

AOH: Absence of heterozygosity
ARIC: Atherosclerosis Risk in Communities
BG: Baylor Genetics
BHCMG: Baylor-Hopkins Center for Mendelian Genomics
CNV: Copy number variant
DD/ID: Developmental delay/intellectual disability
ExAC: The Exome Aggregation Consortium
MAFs: Minor allele frequencies
SNV: Single nucleotide variant
UPD: Uniparental disomy
WES: Whole exome sequencing
WGS: Whole genome sequencing
