# Supplementary material for: Lessons learned from additional research analyses of unsolved clinical exome cases
Source: Genome Med. 2017 Mar 21;9:26. doi: 10.1186/s13073-017-0412-6 (PMC5361813; doi:10.1186/s13073-017-0412-6)
Supplement: Supplementary file 2 — Figures S1, S2, S3, and S4. Computational de novo variant detection from WES data, interactome analysis of USP19, AOH maps for probands with ABCA4 and FBXL4 variants and clinical whole exome management. (PDF 3462 kb) [file 13073_2017_412_MOESM2_ESM.pdf]

S1

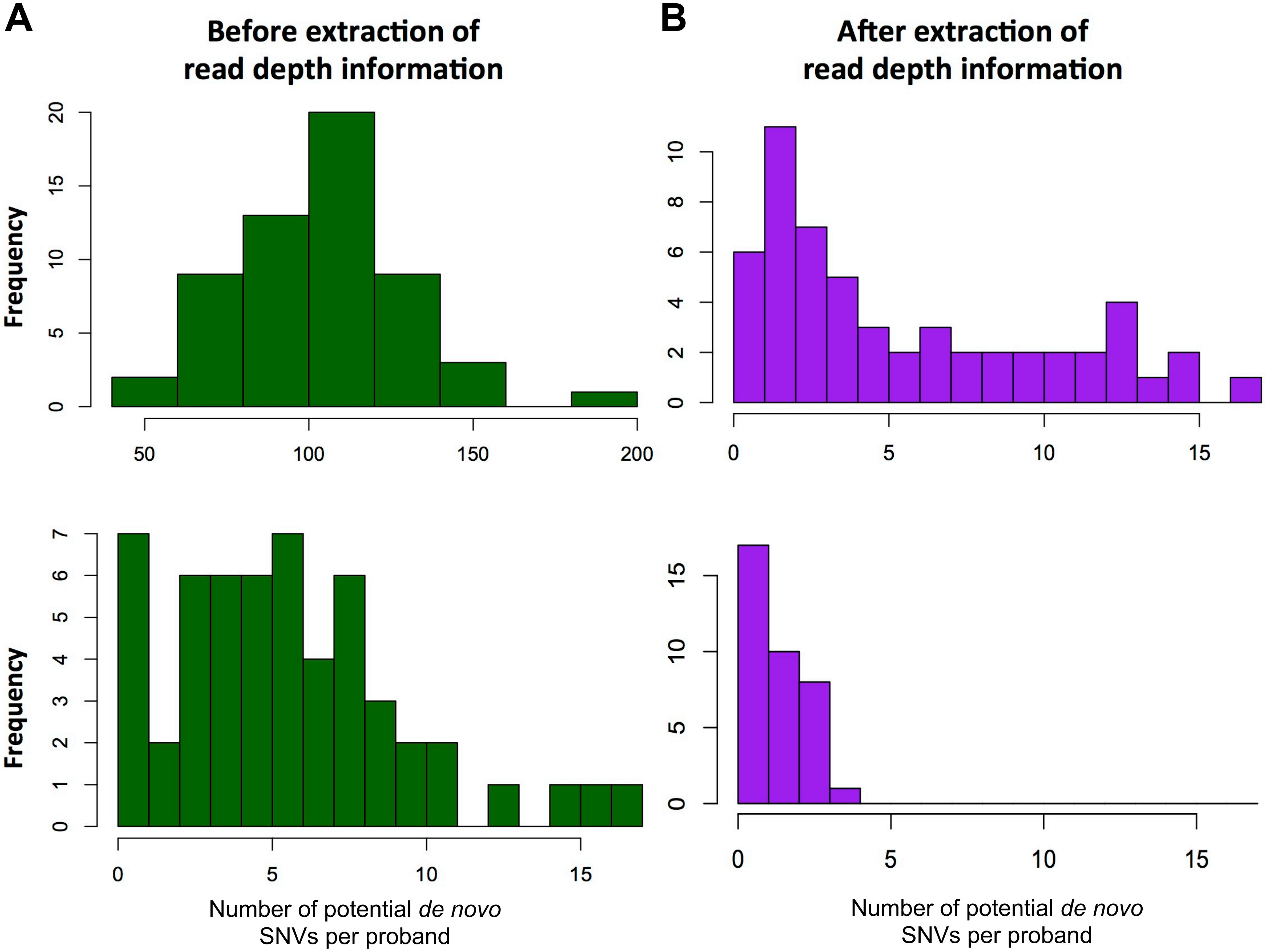
A
Before extraction of read depth information
B
After extraction of read depth information
Frequency
Frequency
Number of potential *de novo* SNVs per proband
Number of potential *de novo* SNVs per proband
0
5
10
15
20
50
100
150
200
0
2
4
6
8
10
0
5
10
15
0
1
2
3
4
5
6
7

S2

http://string-db.org/

Interactome analysis of USP19

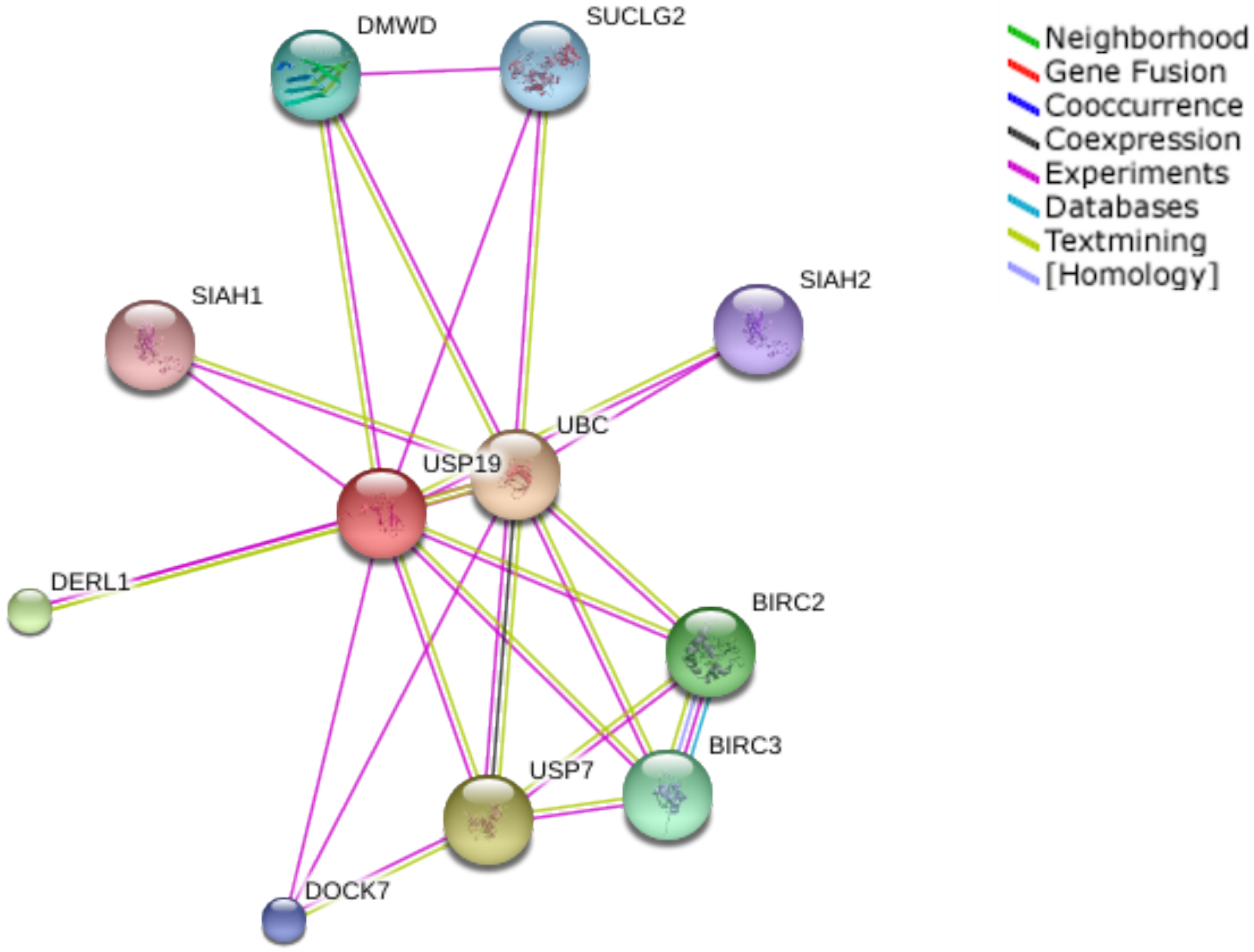

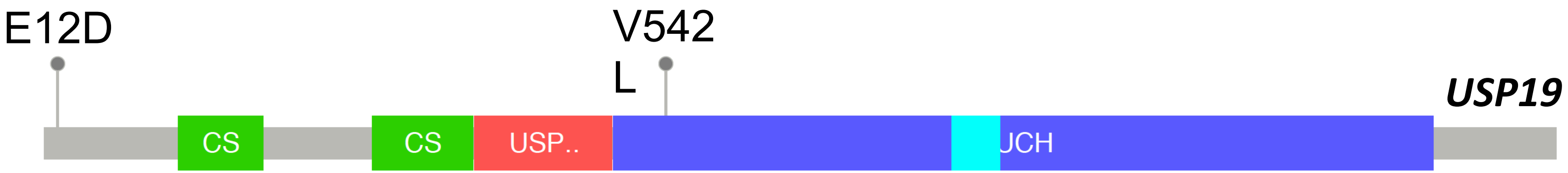

S3

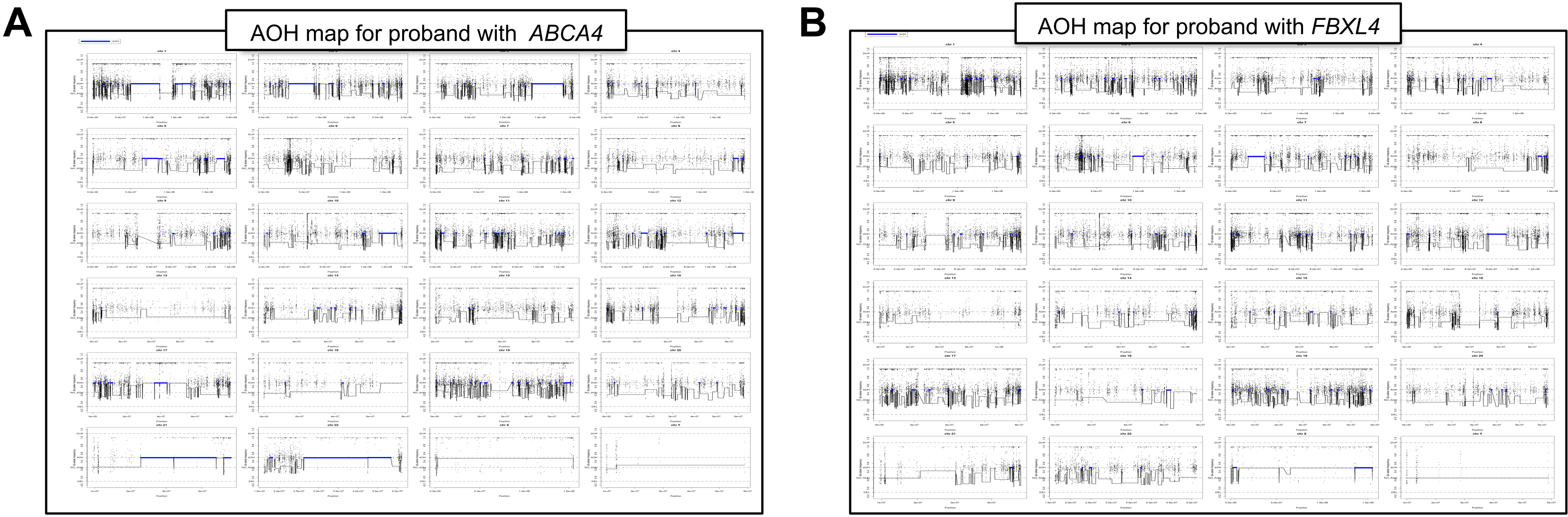

The plot shows the B-allele frequency information (variant/total reads ratio) extracted from exome data VCF files over the genome. Then, we subtracted 0.5 from the signal and then segments with absolute value of the mean signal >0.45 and size >100 kb were detected as AOH regions and displayed in blue. In the presented plot, y axis shows the B-allele frequency (variant/total reads ratio) extracted from exome data VCF files, with each chromosome shown in a separate plot. This B-allele frequency information was then processed using Circular Binary Segmentation (CBS) implemented in the DNAcopy R bioconductor package.

S4

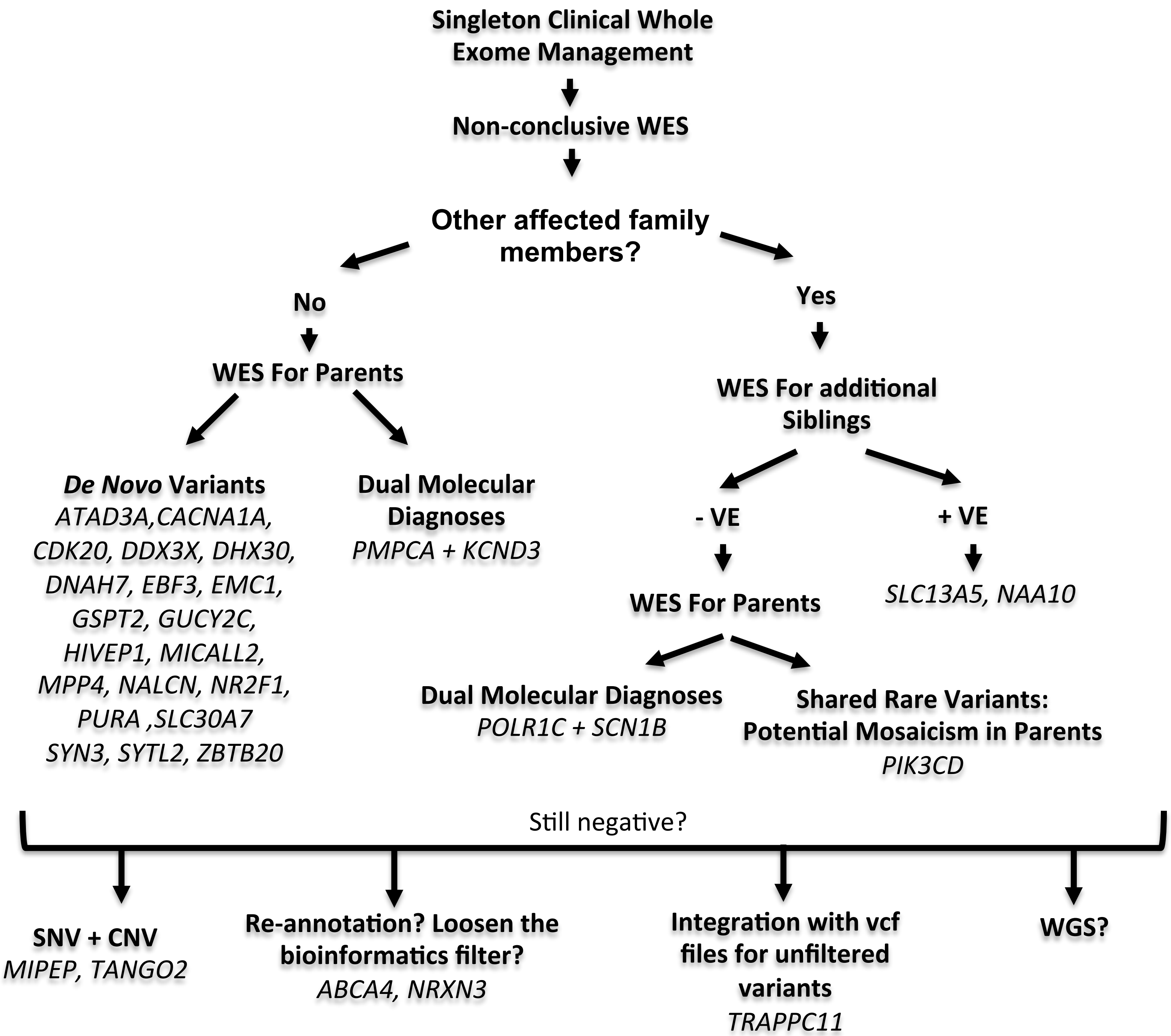
Singleton Clinical Whole Exome Management
Non-conclusive WES
Other affected family members?
No
WES For Parents
De Novo Variants
ATAD3A,CACNA1A, CDK20, DDX3X, DHX30, DNAH7, EBF3, EMC1, GSPT2, GUCY2C, HIVEP1, MICALL2, MPP4, NALCN, NR2F1, PURA ,SLC30A7 SYN3, SYTL2, ZBTB20
Dual Molecular Diagnoses
PMPCA + KCND3
Yes
WES For additional Siblings
- VE
WES For Parents
Dual Molecular Diagnoses
POLR1C + SCN1B
Shared Rare Variants: Potential Mosaicism in Parents
PIK3CD
+ VE
SLC13A5, NAA10
Still negative?
SNV + CNV
MIPEP, TANGO2
Re-annotation? Loosen the bioinformatics filter?
ABCA4, NRXN3
Integration with vcf files for unfiltered variants
TRAPPC11
WGS?
