# Supplementary material for: Lessons learned from additional research analyses of unsolved clinical exome cases
Source: Genome Med. 2017 Mar 21;9:26. doi: 10.1186/s13073-017-0412-6 (PMC5361813; doi:10.1186/s13073-017-0412-6)
Supplement: Supplementary file 4 — Supplemental text. (DOCX 118 kb) [file 13073_2017_412_MOESM4_ESM.docx]

**Supplemental text**

**Lessons Learned from Additional Research Analyses of Unsolved Clinical Exome Cases**

Mohammad K. Eldomery,^1,16,18^ MD; Zeynep Coban-Akdemir,^1,16^ PhD; Tamar Harel,^1,16^ MD, PhD; Jill A. Rosenfeld,^1^ MS, CGC; Tomasz Gambin,^1,2^ PhD; Asbjørg Stray-Pedersen,^3^ MD, PhD; Sébastien Küry,^4^ DVM, PhD; Sandra Mercier,^4,5^ MD, PhD; Davor Lessel,^6^ M.D; Jonas Denecke,^7^ MD; Wojciech Wiszniewski,^1,8^ MD, PhD; Samantha Penney,^1^ MS, CGC; Pengfei Liu,^1,9^ PhD; Weimin Bi,^1,9^ PhD; Seema R. Lalani,^1,8^ MD; Christian P. Schaaf,^1,8,10^ MD, PhD; Michael F. Wangler,^1,8^ MD; Carlos A. Bacino,^1,8^ MD; Richard Alan Lewis,^1,10^ MD, MS; Lorraine Potocki,^1,8^ MD; Brett H. Graham,^1,8^ MD, PhD; John W. Belmont,^1,8^ MD, PhD; Fernando Scaglia,^1,8^ MD; Jordan S. Orange, ^11,12^ MD, PhD; Shalini N. Jhangiani,^13^ MS; Theodore Chiang,^13^ MS; Harsha Doddapaneni,^13^ PhD; Jianhong Hu,^13^ PhD; Donna M. Muzny,^13^ MS; Fan Xia,^1,9^ PhD; Arthur L. Beaudet,^1,9^ MD; Eric Boerwinkle,^13,14^ PhD; Christine M. Eng,^1,9^ MD; Sharon E. Plon,^1,8,11,15^ MD, PhD; V. Reid Sutton,^1,8^ MD; Baylor-Hopkins Center for Mendelian Genomics**,** Richard A. Gibbs,^1,13,^ PhD; Jennifer E. Posey, M.D., PhD;^1^ Yaping Yang,^1,9^ PhD; James R. Lupski,^1,8,11,13,17^ MD, PhD. DSc (hon)

**Author Affiliations:**

^1^Department of Molecular and Human Genetics, Baylor College of Medicine, Houston, TX 77030, USA

^2^ Institute of Computer Science, Warsaw University of Technology, 00-665 Warsaw, Poland

^3^Norwegian National Unit for Newborn Screening, Women and Children’s Division, Oslo University Hospital, 0424 Oslo, Norway

^4^CHU Nantes, Service de Génétique Médicale, 9 quai Moncousu, 44093 Nantes CEDEX 1, France

^5^Atlantic Gene Therapies, UMR1089, Nantes, France

^6^ Institute of Human Genetics, University Medical Center Hamburg-Eppendorf, 20246 Hamburg

^7^Department of Pediatrics, University Medical Center Hamburg-Eppendorf, 20246, Hamburg, Germany

^8^Texas Children’s Hospital, Houston, TX 77030, USA

^9^Baylor Genetics Clinical Laboratories, Baylor College of Medicine, Houston, TX 77030, USA

^10^Jan and Dan Duncan Neurological Research Institute, Texas Children's Hospital, Houston, TX 77030

^11^Department of Pediatrics, Baylor College of Medicine, Houston, TX 77030

^12^Texas Children’s Hospital Center for Human Immuno-Biology, Houston, Texas, USA.

^13^Human Genome Sequencing Center, Baylor College of Medicine, Houston, TX 77030, USA

^14^Human Genetics Center, University of Texas Health Science Center at Houston, Houston, TX 77030, USA

^15^Texas Children's Cancer Center, Texas Children’s Hospital, Houston, TX 7703

^16^These authors contributed equally to this work

^17^Corresponding author

^18^Present Address: Department of Pathology and Laboratory Medicine,

Indiana University School of Medicine, 350 W. 11th Street, Indianapolis, IN 46202, USA.

Dr. James R. Lupski

Department of Molecular and Human Genetics

Baylor College of Medicine

One Baylor Plaza, Room 604B

Houston, TX 77030-3498, USA

Tel: +1-713-798-6530

Fax: +1-713-798-5073

E-mail: jlupski@bcm.edu

^*^Current address:

Department of Pathology and Laboratory Medicine

Indiana University School of Medicine

350 W. 11^th^ Street

Indianapolis, IN, 46202​

E-mail: [m](mailto:jdeppen@iupui.edu)eldomer@iupui.edu

***De novo* changes in candidate genes**

Two individuals had *de novo* variants in two unrelated genes, either of which could be pathogenic: *CDK20* and *HIVEP1* in one proband; and *SLC30A7* and *MICALL2* in another proband. The former presented with seizures, unilateral hearing loss, dysmorphic features, attention deficit hyperactivity disorder (ADHD) and obsessive-compulsive disorder (OCD). A *de novo* stopgain change c.564G>A (p.Trp188*) was identified in cyclin-dependent kinase 20 (*CDK20*, MIM *610076; RefSeq: NM_001039803), important in cell-cycle regulation and transcription. The second *de novo* variant identified in the personal genome of this individual was a c.4089G>C (p.Met1363Ile) variant in human immunodeficiency virus type 1 enhancer-binding protein 1 (*HIVEP1,* MIM *194540; RefSeq: NM_002114), encoding a member of the ZAS gene family of transcription factors important in cell growth.[1] Heterozygous *de novo* loss of function variants in *HIVEP2* were recently identified in individuals with ID, hypotonia, dysmorphic features and behavioral anomalies, making *HIVEP1* a plausible candidate in this individual with a similar phenotype, with a possible additional contribution from *CDK20*.[2]

In a proband who presented with angioedema, chronic abdominal pain, generalized anxiety, migraines, fructose intolerance, and sensitivity to environment and stress challenges, trio WES analysis revealed a *de novo* frameshift deletion c.890delG (p.Ser297Thrfs*8) in synaptotagmin-like protein 2 (*SYTL2*, MIM *612880; RefSeq: NM_032943). Given the reported role of the protein product in the regulation of cytotoxic T cell (CTL) lysosome exocytosis, the phenotype in this subject may potentially be explained by impaired CTL activity caused by the *de novo* variant located in the *SYTL2* gene.[3, 4]

*GSPT2* and *EBF3* each harbored one *de novo* variant in the personal genomes from two separate probands. Both of these genes are located within CNV intervals previously identified in patients with DD/ID, providing additional evidence for a role of these genes in neurodevelopmental phenotypes. In a female with slurred speech and DD/ID, we identified a *de novo* missense variant c.1021G>A (p.Val341Ile) in G1 to S phase transition 2 (*GSPT2*, MIM *300418; RefSeq: NM_018094). *GSPT2*, a single-exon gene located on chromosome X, encodes a GTP-binding protein with an important role in the termination of mammalian protein synthesis through polypeptide chain release factor, eRF3.[8] *GSPT2* has a high level of gene expression in brain and regulates the G1 to S phase transition of the cell cycle.[8] A duplication CNV encompassing *GSPT2* was detected by array comparative genomic hybridization (aCGH) in two affected males with DD/ID.[9] In aggregate, these findings support the contention that gain of function of the gene, through either a *de novo* missense SNV or a gene duplication as has been observed for *PMP22* and Charcot-Marie-Tooth disease, type 1A (CMT1A) (MIM #118220), may cause X-linked DD/ID.

**Biallelic changes in candidate genes**

We identified compound heterozygous changes in Ubiquitin-specific protease 19 (*USP19*) (MIM *614471) in a proband who presented with epileptic encephalopathy, Angelman-like, DD and hypotonia. Recently *USP19* has been shown to be involved in the regulation of *ATXN3* (MIM: 109150), which causes spinocerebellar ataxia-3 (SCA3) by trinucleotide repeat expansion.[10] Furthermore, network analysis shows the interactions of *USP19* with *DOCK7* (MIM *615859), which causes autosomal recessive epileptic encephalopathy as well as with *USP7,* which causes *de novo* autism spectrum disorder.[11, 12] Taken together, we propose that recessive changes in *USP19* could result in a neurodevelopmental disorder.

In addition to the above-mentioned recessive changes in novel genes, a molecular finding with a challenging interpretation was observed in a proband who presented with short stature, severe constipation and macrocephaly, where analyses revealed compound heterozygous changes including a missense variant c.196A>G (p.Lys66Glu) and a splice site variant c.2023+3A>G in neurexin 3 (*NRXN3*, MIM *600567; RefSeq: NM_004796). *NRXN3* CNV deletions have been reported in patients with DD/ID.[13] The neurexin (NRXN) family consists of three members: *NRXN1* (MIM *600565), *NRXN2* (MIM *600566), and *NRXN3*, encoding neuronal cell surface proteins that are highly expressed in brain tissue. This gene family plays a pivotal role in nervous system development.[14] We recently showed that essential genes in fruit fly that have diverged to 2 or more genes in a gene family in human are more likely to represent disease genes.[15] *NRXN1* recessive mutations have been associated with Pitt-Hopkins-like syndrome 2 (MIM #614325), characterized by an abnormal neurological phenotype, facial and skeletal anomalies, gastroesophageal reflux, constipation and pulmonary stenosis.

A homozygous frameshift variant in *ACOT1* (MIM *614313) was identified in a patient who presented with muscle weakness and abnormal muscle biopsy suggestive of a mitochondrial myopathy along with additional features of DD, chronic lung disease and constipation. *ACOT1* encodes acyl-CoA thioesterase 1 (ACOT1), which hydrolyzes acyl-CoA into free fatty acid plus CoA.[16] It is strongly expressed in high-energy demanding tissues such as liver, cardiac, skeletal muscle and brown adipocytes.[16] Peroxisome proliferator-activated receptors (PPARs) and other nutritional factors regulate *ACOT1,* suggesting a role in lipid metabolism and cellular signaling.[17].

**Dual Molecular Diagnosis in Known Genes**

Joint examination of both recessive and *de novo* changes from the trio and quartet WES data aided the establishment of a dual molecular diagnosis in two probands. The first individual presented with epilepsy, hypotonia, ataxia, DD, sensory processing disorder, normal brain MRI, Hirschsprung disease and joint laxity with abnormal mitochondrial function testing (Complex I & III defects). The analysis of trio WES data revealed compound heterozygous variants: c.64C>T (p.Arg22Trp) and c.554G>A (p.Arg185Gln) in peptidase (mitochondrial processing) alpha (*PMPCA*, MIM *613036; RefSeq: NM_015160) as well as a *de novo* variant c.T1153C (p.Ser385Pro) in potassium channel, voltage gated Shal related subfamily D, member 3 (*KCND3*, RefSeq: NM_004980). Recessive variants in *PMPCA* have been recently demonstrated to cause recessive non-progressive cerebellar ataxia and play an important role in the processing and maturation of mitochondrial nuclear encoded proteins inside the mitochondrial matrix.[18] *KCND3* causes an autosomal dominant form of spinocerebellar ataxia type 19 (MIM #607346).[18, 19] We propose that both the *de novo* missense *KCND3* mutation as well as the compound heterozygous variants in *PMPCA* may jointly contribute to this patient's blended phenotype.

In a family with recurrent miscarriages, a male proband presented with neonatal borderline long QT interval and AV block with dilated cardiomyopathy, nystagmus, developmental delay, and polymicrogyria. His sister died at day 6 of life due to multiple organ failure after an episode of ventricular tachycardia, and was also noted to have dysmorphic features and a possible cortical abnormality. Family history was significant for a paternal uncle who died at two years of age secondary to complications related to a congenital heart defect clinically diagnosed as tetralogy of Fallot. His mother had four previous miscarriages, and his father had normal ECG testing. WES data analysis of the two affected siblings and the parents revealed a paternally inherited heterozygous conserved and predicted to be damaging missense variant c.308A>T (p.Asp103Val) in sodium channel, voltage gated, type I beta subunit (*SCN1B*, RefSeq: NM_199037), which is known to cause Brugada syndrome 5 (MIM #612838), segregating in the two affected siblings.[20] In addition, compound heterozygous changes in polymerase (RNA) I polypeptide C (*POLR1C*, MIM *610060; RefSeq: NM_203290) consisting of a paternally inherited conserved and predicted deleterious missense variant c.88C>T (p.Pro30Ser) and a maternally inherited frameshift deletion c.614delG (p.Gly205Alafs*49) were observed in both of the affected siblings. Thirteen homozygous and compound heterozygous mutations in *POLR1C* gene have been recently reported to be causal for leukodystrophy, hypomyelinating syndrome (MIM #616494).[21] A different RNA polymerase gene, *POLR3B,* has been implicated in both hypomyelinating leukodystrophy and polymicrogyria (MIM #614381).[22] Based on these findings, we hypothesize that the presented cardiac phenotype could be explained in part by changes in *SCN1B* gene (although it is inherited from the unaffected father), while compound heterozygous changes in *POLR1C* may lead to the neurological phenotype manifested in these two affected siblings (Additional file 1: Table S1).

**References**

1. Wu LC: **ZAS: C2H2 zinc finger proteins involved in growth and development**. *Gene Expr* 2002, **10**(4):137-152.

2. Srivastava S, Engels H, Schanze I, Cremer K, Wieland T, Menzel M, Schubach M, Biskup S, Kreiss M, Endele S *et al*: **Loss-of-function variants in *HIVEP2* are a cause of intellectual disability**. *Eur J Hum Genet* 2015.

3. Holt O, Kanno E, Bossi G, Booth S, Daniele T, Santoro A, Arico M, Saegusa C, Fukuda M, Griffiths GM: **Slp1 and Slp2-a localize to the plasma membrane of CTL and contribute to secretion from the immunological synapse**. *Traffic* 2008, **9**(4):446-457.

4. Menasche G, Menager MM, Lefebvre JM, Deutsch E, Athman R, Lambert N, Mahlaoui N, Court M, Garin J, Fischer A *et al*: **A newly identified isoform of Slp2a associates with Rab27a in cytotoxic T cells and participates to cytotoxic granule secretion**. *Blood* 2008, **112**(13):5052-5062.

5. Zaltieri M, Grigoletto J, Longhena F, Navarria L, Favero G, Castrezzati S, Colivicchi MA, Della Corte L, Rezzani R, Pizzi M *et al*: **alpha-synuclein and synapsin III cooperatively regulate synaptic function in dopamine neurons**. *J Cell Sci* 2015, **128**(13):2231-2243.

6. Orlic-Milacic M, Kaufman L, Mikhailov A, Cheung AY, Mahmood H, Ellis J, Gianakopoulos PJ, Minassian BA, Vincent JB: **Over-expression of either MECP2_e1 or MECP2_e2 in neuronally differentiated cells results in different patterns of gene expression**. *PLoS One* 2014, **9**(4):e91742.

7. Bozzi Y, Borrelli E: **The role of dopamine signaling in epileptogenesis**. *Front Cell Neurosci* 2013, **7**:157.

8. Hoshino S, Imai M, Mizutani M, Kikuchi Y, Hanaoka F, Ui M, Katada T: **Molecular cloning of a novel member of the eukaryotic polypeptide chain-releasing factors (eRF). Its identification as eRF3 interacting with eRF1**. *J Biol Chem* 1998, **273**(35):22254-22259.

9. Whibley AC, Plagnol V, Tarpey PS, Abidi F, Fullston T, Choma MK, Boucher CA, Shepherd L, Willatt L, Parkin G *et al*: **Fine-scale survey of X chromosome copy number variants and indels underlying intellectual disability**. *Am J Hum Genet* 2010, **87**(2):173-188.

10. He WT, Zheng XM, Zhang YH, Gao YG, Song AX, van der Goot FG, Hu HY: **Cytoplasmic Ubiquitin-Specific Protease 19 (USP19) Modulates Aggregation of Polyglutamine-Expanded Ataxin-3 and Huntingtin through the HSP90 Chaperone**. *PLoS One* 2016, **11**(1):e0147515.

11. Perrault I, Hamdan FF, Rio M, Capo-Chichi JM, Boddaert N, Decarie JC, Maranda B, Nabbout R, Sylvain M, Lortie A *et al*: **Mutations in *DOCK7* in individuals with epileptic encephalopathy and cortical blindness**. *Am J Hum Genet* 2014, **94**(6):891-897.

12. Hao YH, Fountain MD, Jr., Fon Tacer K, Xia F, Bi W, Kang SH, Patel A, Rosenfeld JA, Le Caignec C, Isidor B *et al*: **USP7 Acts as a Molecular Rheostat to Promote WASH-Dependent Endosomal Protein Recycling and Is Mutated in a Human Neurodevelopmental Disorder**. *Mol Cell* 2015, **59**(6):956-969.

13. Vaags AK, Lionel AC, Sato D, Goodenberger M, Stein QP, Curran S, Ogilvie C, Ahn JW, Drmic I, Senman L *et al*: **Rare deletions at the neurexin 3 locus in autism spectrum disorder**. *Am J Hum Genet* 2012, **90**(1):133-141.

14. Ullrich B, Ushkaryov YA, Sudhof TC: **Cartography of neurexins: more than 1000 isoforms generated by alternative splicing and expressed in distinct subsets of neurons**. *Neuron* 1995, **14**(3):497-507.

15. Yamamoto S, Jaiswal M, Charng WL, Gambin T, Karaca E, Mirzaa G, Wiszniewski W, Sandoval H, Haelterman NA, Xiong B *et al*: **A drosophila genetic resource of mutants to study mechanisms underlying human genetic diseases**. *Cell* 2014, **159**(1):200-214.

16. Xia C, Dong R, Chen C, Wang H, Wang DW: **Cardiomyocyte specific expression of Acyl-coA thioesterase 1 attenuates sepsis induced cardiac dysfunction and mortality**. *Biochem Biophys Res Commun* 2015, **468**(4):533-540.

17. Hunt MC, Alexson SE: **The role Acyl-CoA thioesterases play in mediating intracellular lipid metabolism**. *Prog Lipid Res* 2002, **41**(2):99-130.

18. Jobling RK, Assoum M, Gakh O, Blaser S, Raiman JA, Mignot C, Roze E, Durr A, Brice A, Levy N *et al*: ***PMPCA* mutations cause abnormal mitochondrial protein processing in patients with non-progressive cerebellar ataxia**. *Brain* 2015, **138**(Pt 6):1505-1517.

19. Duarri A, Jezierska J, Fokkens M, Meijer M, Schelhaas HJ, den Dunnen WF, van Dijk F, Verschuuren-Bemelmans C, Hageman G, van de Vlies P *et al*: **Mutations in potassium channel *Kcnd3* cause spinocerebellar ataxia type 19**. *Ann Neurol* 2012, **72**(6):870-880.

20. Watanabe H, Koopmann TT, Le Scouarnec S, Yang T, Ingram CR, Schott JJ, Demolombe S, Probst V, Anselme F, Escande D *et al*: **Sodium channel beta1 subunit mutations associated with Brugada syndrome and cardiac conduction disease in humans**. *J Clin Invest* 2008, **118**(6):2260-2268.

21. Thiffault I, Wolf NI, Forget D, Guerrero K, Tran LT, Choquet K, Lavallee-Adam M, Poitras C, Brais B, Yoon G *et al*: **Recessive mutations in *POLR1C* cause a leukodystrophy by impairing biogenesis of RNA polymerase III**. *Nat Commun* 2015, **6**:7623.

22. Jurkiewicz E, Dunin-Wasowicz D, Gieruszczak-Bialek D, Malczyk K, Guerrero K, Gutierrez M, Tran L, Bernard G: **Recessive Mutations in *POLR3B* Encoding RNA Polymerase III Subunit Causing Diffuse Hypomyelination in Patients with 4H Leukodystrophy with Polymicrogyria and Cataracts**. *Clin Neuroradiol* 2015.

**Web Resources**

Atherosclerosis Risk in Communities Study, <http://www2.cscc.unc.edu/aric/>

Baylor Genetics Laboratories

https://www.bcm.edu/research/medical-genetics-labs/

cBioPortal for Cancer Genomics, <http://www.cbioportal.org/index.do>

Exome Aggregation Consortium (ExAC), <http://exac.broadinstitute.org>

Ensembl, http://www.ensembl.org/

GeneMatcher, <https://genematcher.org/>

HMZDelFinder, <https://github.com/BCM-Lupskilab/HMZDelFinder>

Mutation taster, <http://www.mutationtaster.org/>

NHLBI Exome Sequencing Project (ESP) Exome Variant Server, <http://evs.gs.washington.edu/EVS/>

Online Mendelian Inheritance in Man (OMIM), <http://www.omim.org/>

PhenoDB, <https://mendeliangenomics.org/>

PolyPhen2, <http://genetics.bwh.harvard.edu/pph2/>

# PubMed, http://www.ncbi.nlm.nih.gov/pubmed

RefSeq, <http://www.ncbi.nlm.nih.gov/refseq>

SIFT, <http://sift.jcvi.org/>

1000 Genomes, <http://www.1000genomes.org/>

UCSC Genome Browser, <http://genome.ucsc.edu/index.html>
